# Computer science work and interest profiles: stereotype vs. realities

**DOI:** 10.1038/s41598-023-47963-3

**Published:** 2023-12-11

**Authors:** Rong Su, Dan J. Putka, James Rounds

**Affiliations:** 1 Department of Management and Entrepreneurship, Tippie College of Business, University of Iowa, Iowa City, IA 52242 USA; 2 Human Resources Research Organization, Alexandria, VA 22314 USA; 3 Department of Psychology and Educational Psychology, University of Illinois, Champaign, IL 61820 USA

**Keywords:** Psychology, Human behaviour

**arising from**: J.E. McChesney et al.; *Scientific Reports* 10.1038/s41598-022-09522-0 (2022).

## Introduction

Increasing the representation of women in science, technology, engineering, and mathematics (STEM) is critically important for the effective utilization of human capital and the sustained competitiveness of these fields. McChesney et al.^1^ demonstrated the discrepancy between the career interests of individuals they believed to be currently in or aspiring to computer science (CS) occupations and the average Occupational Interest Profile (OIP) for those occupations from the U.S. Department of Labor’s Occupational Information Network (O*NET). They cautioned that stereotypical descriptions of CS career interests could deter women from entering the field. While we share their view on the need to represent interest diversity within CS (and STEM occupations in general), we see the critical need to clarify the nature of the OIPs and point out that McChesney et al.’s analyses and conclusions were based on a heterogeneous set of jobs rather than CS occupations. Importantly, we suggest evidence-based practices for broadening the participation of women in STEM.

## OIPs are not stereotypical descriptions of occupations

McChesney et al.^1^ characterized OIPs from O*NET as “inferred interests of job incumbents” and framed OIPs as “stereotypical depictions” of occupations. Despite containing the word “interest,” an OIP does not measure interests of job incumbents within an occupation; nor does it measure the stereotype about an occupation. Rather, an OIP was developed to reflect the extent to which Holland’s^2^ RIASEC work environments are descriptive and characteristic of core tasks and activities typically performed in an occupation^3^. These tasks and activities were also identified via a rigorous, systematic data collection process^4^. This distinction is important, because the purpose of the OIPs is to provide information *at the occupational level* that helps individuals understand the type of work involved in different careers so as to facilitate career exploration. Thus, the OIP for CS describes common tasks and activities incumbents do in CS, not the people in these occupations who may have diverse interests. By no means does the OIP represents the stereotypical interest profile of CS professionals. Characterizing the OIPs as stereotypical depictions of occupations may discourage individuals from using occupational information from O*NET, which provides the nation’s most reliable and useful resource for career exploration and job search.

## Heterogeneity of jobs classified as computer science occupations

The focus of McChesney et al.^1^ was on what they called “CS professions”. However, their definition of CS professions was not well specified (“those requiring some form of experience in computing”) and the classification process was conducted by Amazon Mechanical Turk workers with no background or training in occupational classification. As a result, 21 out of the 46 job titles that were classified as “CS professions” in McChesney et al.^1^ belonged instead to 11 other occupational groups that are very different in nature, ranging from business/finance (e.g., “Marketing Specialists”) to arts/design (e.g., “Graphic designers”) to service occupations (e.g., “Gaming Supervisors”). Though some of these jobs may be tangentially related to computer science because of the use of computers in daily work (e.g., marketing specialists, graphic designers, desktop publishers), others are not related to computer science at all (e.g., gaming supervisors, which refer to personnel who supervise and coordinate activities of workers in gambling services).

In Table 1, we list these 21 jobs and their corresponding occupational categories based on the Bureau of Labor Statistics’ Standard Occupational Classification (SOC) system^5,6^. These jobs employed 344 out of the 500 participants whose interest scores were analyzed by McChesney et al.^1^. In other words, nearly 70% of the participants worked in jobs outside the field of computer science. Each of these jobs has an OIP that is distinctively different from that of CS. Many of them, including the examples highlighted above, have higher levels of Artistic, Enterprising, and/or Social characteristics. Thus, the latent interest profiles McChesney et al.^1^ developed based on this occupationally diverse sample reflected not the interests of “computer scientists” but the interests of individuals employed in a heterogenous set of jobs. Coincidentally, about 70% of their participants displayed interest profiles that deviated from what was deemed normative for CS job incumbents. For example, with a sizable proportion of the 500 participants employed in arts/design-related jobs, it is not surprising that an Artistic latent interest profile was well represented in the data. Therefore, the diverse interest profiles reported by McChesney et al.^1^ were, at least in part, due to the inclusion of a heterogeneous set of non-CS jobs and workers in the analyses.

**Table 1:** Classification of job titles in McChesney et al. based on the SOC.

Job title	Number of participants in job	O*NET-SOC 2019 code for job	SOC category for job
Market Research Analysts and Marketing Specialists	109	13-1161.00	13-0000 Business and Financial Operations Occupations
Graphic Designers	69	27-1024.00	27-0000 Arts, Design, Entertainment, Sports, and Media Occupations
Computer Hardware Engineers	24	17-2061.00	17-0000 Architecture and Engineering Occupations
Intelligence Analysts	24	33-3021.06	33-0000 Protective Service Occupations
Search Marketing Strategists	16	13-1161.01	13-0000 Business and Financial Operations Occupations
Securities and Commodities Traders	16	No exact match; closest job code: 13-2051.00	13-0000 Business and Financial Operations Occupations
Desktop Publishers	12	43-9031.00	43-0000 Office and Administrative Support Occupations
Computer, Automated Teller, and Office Machine Repairers	11	49-2011.00	49-0000 Installation, Maintenance, and Repair Occupations
Data Entry Keyers	10	43-9021.00	43-0000 Office and Administrative Support Occupations
Quality Control Analysts	9	19-4099.01	19-0000 Life, Physical, and Social Science Occupations
Logistics Managers	9	No exact match; closest job code: 13-1081.00	13-0000 Business and Financial Operations Occupations
Air Traffic Controllers	9	53-2021.00	53-0000 Transportation and Material Moving Occupations
Sound Engineering Technicians	8	27-4014.00	27-0000 Arts, Design, Entertainment, Sports, and Media Occupations
Audio-Visual and Multimedia Collections Specialists	4	No exact match; closest job code: 27-4011.00	27-0000 Arts, Design, Entertainment, Sports, and Media Occupations
Financial Quantitative Analysts	3	13-2099.01	13-0000 Business and Financial Operations Occupations
Logistics Analysts	3	13-1081.02	13-0000 Business and Financial Operations Occupations
Quality Control Systems Managers	3	11-3051.01	11-0000 Management Occupations
Computer Numerically Controlled Machine Tool Programmers, Metal and Plastic	2	51-9162.00	51-0000 Production Occupations
Robotics Technicians	1	17-3024.01	17-0000 Architecture and Engineering Occupations
Microsystems Engineers	1	17-2199.06	17-0000 Architecture and Engineering Occupations
Gaming supervisors	1	No exact match; closest job code: 39-1013.00	39-0000 Personal Care and Service Occupations
Total number of jobs in McChesney et al. that belong to other occupational groups based on the SOC: 21 (45.7%) Total number of participants from these jobs: 344 (68.8%)			

Computer science occupations belong to SOC category 15-0000 Computer and Mathematical Occupations.

## Addressing key questions: interest diversity within occupations

McChesney et al.’s^1^ characterization of OIPs and CS occupations notwithstanding, they did pose an excellent question—that is, could individual career interests diverge from characteristics of the occupational environments they are in? The answer is yes. Research across over 100 occupations in the U.S. demonstrated high levels of diversity in individual interests *within* occupations^7^. In other words, although career interests are major drivers of individuals’ career choices and individuals are likely found in occupations that match their interests than not, many will have interests different from the types of interests their occupations typically fulfill. It is well expected that all individuals within an occupation will not share the same interest profile. Meanwhile, the level of interest diversity has also been shown to vary significantly across occupations^7^, with fine artists and physicists being some of the most homogenous and dietitians and chiropractors being some of the most heterogeneous. A more nuanced and comprehensive picture of interests in CS and other STEM fields would indeed be important, but it would need to be drawn from large, representative samples.

If interest diversity is expected among members within an occupation, is it still valid to provide career guidance to individuals based on the comparison of their career interests and characteristic descriptions of an occupational environment, such as the OIP? We argue that it is. Over a century of research on career interests and many large-scale studies and meta-analyses have shown that interest congruence—the extent to which individual interests match the characteristics of their occupational environments—is predictive of individuals’ job satisfaction, job performance, persistence on the job, and career success^8–10^. Without accurately understanding and characterizing occupational environments, individuals lack information to explore and choose occupations in which they are most likely to be satisfied and successful and most likely to stay. To discuss the diversity of individual interests within an occupation without considering the importance of interest congruence for these work outcomes can be misleading. In this regard, occupational information provided by databases such as O*NET and U.K.’s National Career Services and assessments of interest congruence based on such information offer invaluable guidance, rather than reinforcing stereotypes.

## Closing the gender gap: a path forward

Considering substantial gender differences in career interests—particularly differences along the things-people dimension with females expressing stronger interests in working with people^11,12^, how do we address the fact that fewer girls and women may find their interests congruent with CS and other STEM fields? To broaden participation in STEM, one promising path forward is *relational job design*^13^. Organizations may incorporate people-oriented tasks and activities in the design of specific jobs and emphasize the prosocial impact of the jobs when recruiting candidates. Similarly, STEM educational programs can incorporate and highlight opportunities for people-oriented tasks and activities as a way to attract more girls and women with social interests and communal values^14^. At the individual level, initiatives designed to cultivate girls’ interest in STEM solve one piece of gender gap puzzle. Other potential interventions could aim to make science and math activities personally relatable, showcase real-world applications, highlight successful female role models, and help girls develop strong self-concepts in these areas^15^. These interventions would also need to occur early and be widely accessible to the public. An excellent example is “SciGirls”, a recent television series funded by the U.S. National Science Foundation, that features young girls performing hands-on scientific experiments with female scientist mentors. Programs like this supplement the rich information about occupational environments provided by databases such as O*NET to paint a realistic and yet accessible picture of what inclusive STEM careers are like.

## References

[1] McChesney JE, Behrend TS, Glosenberg A (2022). Stereotypical descriptions of computer science career interests are not representative of many computer scientists. Sci. Rep..

[2] Holland JL (1997). Making Vocational Choices: A Theory of Vocational Personalities and Work Environments.

[3] Rounds, J., Smith, T., Hubert, L., Lewis, P., & Rivkin, D. *Development of Occupational Interest Profiles (OIPs) for the O*NET*. http://www.onetcenter.org/reports/OIP.html (National Center for O*NET Development, 1999).

[4] Dierdorff, E. C., & Norton, J. J*. Summary of Procedures for O*NET Task Updating and New Task Generation*. https://www.onetcenter.org/reports/TaskUpdating.html (National Center for O*NET Development, 2011).

[5] U.S. Bureau of Labor Statistics. *2018 Standard Occupational Classification System*. https://www.bls.gov/soc/2018/major_groups.htm (2018).

[6] Gregory, D., Lewis, P., Frugoli, P., & Nallin, A. *Updating the O*NET-SOC Taxonomy: Incorporating the 2018 SOC Structure*. https://www.onetcenter.org/reports/Taxonomy2019.html (National Center for O*NET Development, 2019).

[7] Nye CD, Perlus JG, Rounds J (2018). Do ornithologists flock together? Examining the homogeneity of interests in occupations. J. Vocat. Behav..

[8] Hoff KA, Song QC, Wee CJ, Phan WMJ, Rounds J (2020). Interest fit and job satisfaction: A systematic review and meta-analysis. J. Vocat. Behav..

[9] Nye CD, Su R, Rounds J, Drasgow F (2017). Interest congruence and performance: Revisiting recent meta-analytic findings. J. Vocat. Behav..

[10] Su R (2020). The three faces of interests: An integrative review of interest research in vocational, organizational, and educational psychology. J. Vocat. Behav..

[11] Su R, Rounds J, Armstrong PI (2009). Men and things, women and people: A meta-analysis of sex differences in interests. Psychol. Bull..

[12] Su R, Rounds J (2015). All STEM fields are not created equal: People and things interests explain gender disparities across STEM fields. Front. Psychol..

[13] Grant AM (2007). Relational job design and the motivation to make a prosocial difference. Acad. Manag. Rev..

[14] Diekman AB, Clark EK, Johnston AM, Brown ER, Steinberg M (2011). Malleability in communal goals and beliefs influences attraction to stem careers: Evidence for a goal congruity perspective. J. Pers. Soc. Psychol..

[15] Wang MT, Degol JL (2017). Gender gap in science, technology, engineering, and mathematics (STEM): Current knowledge, implications for practice, policy, and future directions. Educ. Psychol. Rev..

